# ESOPHAGEAL LICHEN PLANUS: A CASE REPORT AND REVIEW OF LITERATURE

**DOI:** 10.4103/0019-5154.39738

**Published:** 2008

**Authors:** K S Madhusudhan, Raju Sharma

**Affiliations:** *From Department of Radiology, All India Institute of Medical Sciences, New Delhi, India*

**Keywords:** *Dysphagia*, *esophageal stricture*, *lichen planus*

## Abstract

Lichen planus is a rare cause of esophagitis and esophageal stricture. It is invariably associated with oral mucosal involvement and the diagnosis has to be considered in these patients who present with dysphagia. We present a case of esophageal stricture secondary to lichen planus.

## Introduction

Benign long segment esophageal strictures are caused by a number of conditions, including corrosive intake, gastroesophageal reflux, radiation and nasogastric intubation. In addition, a number of dermatologic conditions also cause esophageal strictures. These include epidermolysis bullosa, pemphigoid, pemphigus vulgaris and lichen planus.^1^

Lichen planus is a dermatologic condition of unknown etiology involving the skin, nails and mucosal membranes. The skin lesions are characterized by shiny, violaceous, flat papules, of varying size. It typically remits and recurs, with recurrences lasting years. They are mainly localized to the front of the wrists, lumbar region and around the ankles. Mucous membrane lesions occur in 30-70% of cases and may be found without evidence of skin lesions.^2^ They consist of white plaques, erosions and rarely ulcerations and involve the buccal mucosa, tongue, genitalia and anus.^2^

Esophageal involvement is rare^3^,^4^ with just over 20 cases reported in the literature. The rarity and unfamiliarity of the disease causes delay in early diagnosis of the disease. We report a case of oral lichen planus, controlled on steroids who developed long segment esophageal stricture secondary to involvement by lichen planus.

## Case Report

A 55-year-old female presented with history of progressively increasing dysphagia for solid foods for the past six months. She was a known case of oral lichen planus and was on treatment with steroids for the past five years. The patient did not have any history suggestive of gastroesophageal reflux or any history of intake of drugs known to cause esophagitis.

**Fig. 1:**
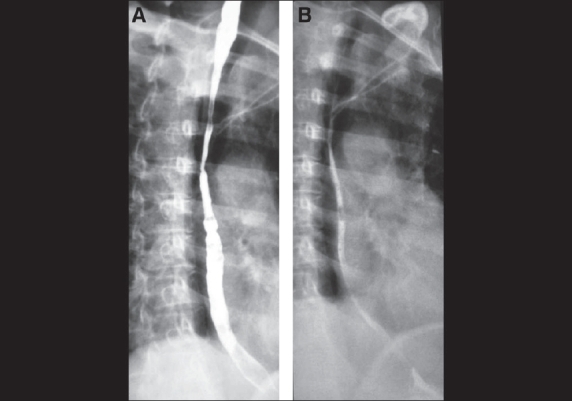
Barium swallow picture in full phase (A) and mucosal relief phase (B) shows smooth long segment stricture involving the upper and mid-thoracic esophagus. Note the tertiary contractions in the lower half of the esophagus

Barium swallow was done which showed a long segment benign stricture of the upper thoracic esophagus [Fig. 1A and B]. Subsequently endoscopy showed narrowing of upper thoracic esophagus and the scope could not be negotiated distally. The mucosa proximal to the stricture showed erosions. Biopsy taken from this region showed inflammatory cell infiltrates with lymphocytes. Based on the history, barium and endoscopic appearance and the biopsy finding, a diagnosis of esophageal lichen planus was made. Patient underwent endoscopic dilatation in three sittings and her symptoms improved.

## Discussion

Esophageal lichen planus is a rare condition,^3^,^4^ although its exact incidence is not known. In an endoscopic study of the esophagus in patients with lichen planus by Dickens *et al.*^5^ it was found that about a quarter of the patients (5 of 19) had esophageal involvement. Singh *et al.* endoscopically studied the upper gastrointestinal tract of 21 patients, in whom they found mucosal lesions in 76% (16) patients; however, none had esophageal stricture.^6^ Esophageal involvement is characterized by lesions in the form of peeling of the friable mucosa, white plaques, ulcers, erosions and stricture formation^7^ and typically affects the upper and mid-esophagus sparing the gastroesophageal junction, unlike reflux esophagitis.^4^

It is typically seen in the age group 44-79 years and all the reported cases are females.^4^,^7^ All patients had buccal involvement. Symptomatic patients present with progressively increasing dysphagia and an esophagoscopy should be performed in all patients of lichen planus with gastrointestinal symptoms.

Barium swallow may show a long segment smooth stricture involving the upper and mid-thoracic esophagus; such an appearance is nonspecific and may be seen in gastroesophageal reflux disease, corrosive stricture or after intake of drugs like nonsteroidal anti-inflammatory drugs. Our patient did not have any history of drug intake and the biopsy material did not show any infectious agent. She did not have any reflux symptoms. Also, involvement of the upper esophagus is atypical of reflux-induced esophagitis.

The pathological features of esophageal lichen planus resemble those of oral lesions rather than the cutaneous lesions. All sites of involvement show a band-like inflammatory infiltrate with a predominance of mature T cells and basal layer degeneration, including characteristic Civatte bodies.^7^ The submucosal lymphocytic infiltration though consistent with lichen planus, is nonspecific and is seen in patients on drugs like gold, thiazides, anti-malarials, in infectious or pill-induced esophagitis and occasionally in gastroesophageal reflux disease.^7^

Proximal esophageal stricture, mural infiltration by lymphocytes and the presence of oral lesions favored the diagnosis of esophageal lichen planus. Failure to make a diagnosis prevents early management of the condition. Esophageal lichen planus has a propensity for chronicity and requires topical or systemic therapy with corticosteroids, retinoids, cyclosporine and griseofulvin.^8^ Dilatation is required once stricture has developed, although endoscopic manipulation may exacerbate the oral lesions.^9^

Oral lichen planus has a very low malignant potential of less than 1%. However, no cases of malignant transformation of esophageal lesions have been reported, though it poses a theoretical risk.^9^

To conclude, lichen planus is a rare cause of esophageal stricture, which is often mistaken for other common causes like drug-induced or reflux disease. Dermatologic disease must be considered in patients with proximal esophageal involvement, especially in those with either cutaneous or mucosal lesions.
